# Treatment of aggressive posterior retinopathy of prematurity accompanied by nasolacrimal duct obstruction with purulent discharge: A case report

**DOI:** 10.1002/ccr3.9533

**Published:** 2024-11-05

**Authors:** Ehsan Namvar, Alireza Attar

**Affiliations:** ^1^ Department of Ophthalmology, Poostchi Ophthalmology Research Center, School of Medicine Shiraz University of Medical Sciences Shiraz Iran

**Keywords:** bevacizumab injection, intravitreal injection, nasolacrimal duct obstruction, retinopathy of prematurity

## Abstract

This case demonstrates that probing and 10% povidone‐iodine irrigation of the nasolacrimal duct prior to intravitreal bevacizumab injection can safely reduce the risk of endophthalmitis in infants with aggressive posterior retinopathy of prematurity and nasolacrimal duct obstruction. This method provides a viable treatment alternative when laser therapy alone is insufficient.

## 1 BACKGROUND

Retinopathy of prematurity (ROP) is a condition characterized by abnormal development of retinal blood vessels in premature infants, leading to impaired growth and functionality of these vital ocular structures.^1^ Aggressive posterior retinopathy of prematurity (APROP) manifests as a notably severe form of the condition, featuring prominent plus disease, a distinctive presentation of flat neovascularization primarily in zone 1 or posterior zone 2 of the retina, intraretinal shunting, significant hemorrhage, and an accelerated trajectory toward retinal detachment.^2^ Addressing posterior ROP, typically Zone I has proved to be challenging. Despite laser or cryotherapy, retinal detachment risk remains high.^3^, ^4^ Previously, laser treatment was common, but anti‐VEGF, particularly for Type 1 ROP, is now prevalent. Past approaches caused retinal damage; anti‐VEGF fosters nondestructive vascular growth. Late‐stage retinal detachment demands scleral buckling and vitrectomy.^5^, ^6^ Endophthalmitis, a severe infection, stands as a dreaded outcome of intraocular surgery, often resulting in irreversible vision impairment.^7^ Postoperative endophthalmitis is mostly attributed to gram‐positive bacteria, notably Staphylococcus epidermidis and Staphylococcus aureus, commonly found on the eyelid and conjunctiva.^8^ The lacrimal system typically prevents bacterial colonization of the eyelids and conjunctiva by facilitating drainage. However, an obstructed nasolacrimal duct can trap bacteria within the lacrimal sac, leading to reflux onto the ocular surface. This bacterial reflux poses a significant infection risk during intraocular surgery, potentially contaminating the procedure.^9^ Nasolacrimal duct obstruction (NLDO) is the most common disorder of the lacrimal system in newborns, affecting 6%–20%. It typically presents within the first weeks of life with symptoms such as excessive tearing and ocular discharge.^10^

Povidone‐iodine (PI) is a prophylactic treatment to prevent endophthalmitis in cataract surgery.^11^ PI ocular irrigation during cataract surgery decreases the bacterial detection rate to 0%.^12^ In addition, PI has been used in different ophthalmic procedures, especially for intravitreal injection.^13^, ^14^, ^15^, ^16^

The aim of this study was to present a treatment modality for patients with ROP who require intraocular injection despite nasolacrimal duct obstruction with purulent discharge.

## 2 CASE PRESENTATION

### 2.1 Case history

We present a case study of a one‐month‐old neonate born at 30 weeks of gestation due to premature rupture of membranes (PROM), with an Apgar score of 8 and a birth weight of 1745 grams. The neonate spent 12 days in the neonatal intensive care unit (NICU) due to complications associated with prematurity and required nasal oxygen supplementation for 5 days. The neonate had no history of sepsis and did not receive any blood transfusions. Despite the absence of a family history of ocular diseases and normal visual development in both parents and siblings, the neonate was referred to Poostchi Eye Clinic at 34 weeks of gestational age for evaluation due to the risks associated with prematurity.

## 3 METHODS

The diagnosis included nasolacrimal duct obstruction with significant purulent discharge from the right eye, alongside aggressive posterior ROP in both eyes. The neonate was urgently referred to Khalili Hospital. In this case, ROP was at posterior zone 2, but due to nasolacrimal duct obstruction and purulent discharge and subsequently increased risk of endophthalmitis after anti‐VEGF injection, laser treatment was chosen first. Therefore, 360° laser ablation therapy was performed in both eyes without anti‐VEGF injection due to the presence of nasolacrimal duct obstruction and purulent discharge. Five days later, the patient's condition worsened. On examination, arterial tortuosity and venous dilation of the posterior vessels with associated hemorrhage were observed, along with peripheral retinal detachment. Fortunately, the macula remained attached. These findings were consistent with stage 4a ROP in posterior zone 2. As a result, we performed lacrimal duct probing and irrigation of the nasolacrimal duct with 10% PI, followed by an intravitreal injection of bevacizumab (0.625 mg/0.025 mL). One week after the injection, the hemorrhage had decreased, and the retina had reattached.

## 4 CONCLUSION

This case study presents a newborn who faced two challenges: 1‐bilateral APROP due to his very early birth at 30 weeks which needed an intravitreal anti‐VEGF injection, 2‐Nasolacrimal duct obstruction and purulent discharge that increases the risk of endophthalmitis. In this challenging case, despite the infection and risk of endophthalmitis, the neonate needed treatment for the eye disease; therefore, only laser ablation therapy was done first. However, 5 days later, the patient's condition was aggravated and he developed hemorrhage and retinal elevation. At that moment, the intravitreal anti‐VEGF injection was mandatory to rescue the eye, so probing and irrigation of the nasolacrimal duct with PI 10% was done immediately. Thereafter, intravitreal bevacizumab (IVB) was injected into both eyes. However, further larger studies are needed to confirm the safety and efficacy of this approach.

## 5 DISCUSSION

This is the first reported case of an infant with both aggressive posterior ROP and nasolacrimal duct obstruction was treated with probing, irrigation with PI 10%, injections of IVB into both eyes and laser therapy. NLDO and purulent discharge cause tear stasis, increased bacterial load, and retrograde infection, elevating endophthalmitis risk, especially during ocular surgeries.^17^ Managing NLDO is crucial, and PI offers broad‐spectrum efficacy, rapid action, and safety, reducing infection risk and tissue irritation without promoting resistance.^18^

Similarly, Shimada et al.'s study indicated that 0.25% PI irrigation and surgical mask reduced the endophthalmitis rate following intravitreal injections.^13^ However, none of these studies simultaneously evaluated the risk of endophthalmitis in the presence of active infection.

It is obvious that in the presence of nasolacrimal duct obstruction with purulent discharge, laser treatment is a better choice than anti‐VEGF injection in patients with ROP who needs treatment unless the patient became aggravated or has stage 4a ROP.

Vitrectomy is another treatment choice in stage 4a ROP, but nasolacrimal duct obstruction with purulent discharge increases the risk of endophthalmitis after vitrectomy too. In‐addition vitrectomy may contribute to several complications including cataract, retinal detachment and hemorrhage.^19^

Laser treatment combined with and anti‐VEGF therapy is a safe and effective non‐surgical treatment of stage 4A ROP, that may decrease the need for vitrectomy.^20^ In according to these results, we chose nasolacrimal duct irrigation with povodine iodine and then anti‐VEGF injection before selecting vitrectomy.

The limitations of our study included its single‐case nature and the lack of long‐term follow‐up.

Although this case study suggests that IVB injections immediately after probing and PI 10% irrigation are safe and effective for treating this type of eye disease, more studies with larger sample sizes are needed to be conducted in the future. Probing and PI 10% irrigation of the nasolacrimal duct and ocular surface is a safe and effective method to prevent endophthalmitis following IVB injection in a patient with aggressive posterior ROP who does not respond to laser monotherapy.

## AUTHOR CONTRIBUTIONS

**Ehsan Namvar:** Project administration. **Alireza Attar:** Project administration.

## FUNDING INFORMATION

None declared.

## CONFLICT OF INTEREST STATEMENT

The authors declare no competing interests.

## ETHICS STATEMENT

ethic approval and consent to oarticipate changed to ethic statement.

## CONSENT

Written informed consent was obtained from the patient to publish this report in accordance with the journal's patient consent policy.

## Data Availability

The datasets used and/or analyzed during the current study are available from the corresponding author on reasonable request.
